# Atmospheric and Room Temperature Plasma (ARTP) Mutagenesis Improved the Anti-MRSA Activity of *Brevibacillus* sp. SPR20

**DOI:** 10.3390/ijms241512016

**Published:** 2023-07-27

**Authors:** Nuttapon Songnaka, Monthon Lertcanawanichakul, Albert Manggading Hutapea, Mudtorlep Nisoa, Sucheewin Krobthong, Yodying Yingchutrakul, Apichart Atipairin

**Affiliations:** 1 School of Pharmacy, Walailak University, Nakhon Si Thammarat 80161, Thailand; nuttapon.so@wu.ac.th; 2 Drug and Cosmetics Excellence Center, Walailak University, Nakhon Si Thammarat 80161, Thailand; 3 School of Allied Health Sciences, Walailak University, Nakhon Si Thammarat 80161, Thailand; lmonthon@wu.ac.th; 4 Faculty of Science, Universitas Advent Indonesia, Bandung 40559, Indonesia; amhutapea@unai.edu; 5 School of Science, Walailak University, Nakhon Si Thammarat 80161, Thailand; nmudtorl@wu.ac.th; 6 Center of Excellence in Plasma Science and Electromagnetic Waves, Walailak University, Nakhon Si Thammarat 80161, Thailand; 7 Center of Excellence in Natural Products Chemistry (CENP), Department of Chemistry, Faculty of Science, Chulalongkorn University, Bangkok 10330, Thailand; sucheewin82@gmail.com; 8 National Center for Genetic Engineering and Biotechnology, National Science and Technology Development Agency, Pathum Thani 12120, Thailand; yodying.yin@nstda.or.th

**Keywords:** atmospheric and room temperature plasma (ARTP), anti-MRSA activity, *Brevibacillus* sp., mutagenesis, proteomic analysis, strain improvement

## Abstract

*Brevibacillus* sp. SPR20 produced potentially antibacterial substances against methicillin-resistant *Staphylococcus aureus* (MRSA). The synthesis of these substances is controlled by their biosynthetic gene clusters. Several mutagenesis methods are used to overcome the restriction of gene regulations when genetic information is absent. Atmospheric and room temperature plasma (ARTP) is a powerful technique to initiate random mutagenesis for microbial strain improvement. This study utilized an argon-based ARTP to conduct the mutations on SPR20. The positive mutants of 40% occurred. The M27 mutant exhibited an increase in anti-MRSA activity when compared to the wild-type strain, with the MIC values of 250–500 and 500 μg/mL, respectively. M27 had genetic stability because it exhibited constant activity throughout fifteen generations. This mutant had similar morphology and antibiotic susceptibility to the wild type. Comparative proteomic analysis identified some specific proteins that were upregulated in M27. These proteins were involved in the metabolism of amino acids, cell structure and movement, and catalytic enzymes. These might result in the enhancement of the anti-MRSA activity of the ARTP-treated SPR20 mutant. This study supports the ARTP technology designed to increase the production of valuable antibacterial agents.

## 1. Introduction

Infectious disease is one of the leading causes of mortality throughout the world. It involves the invasion of pathogenic microorganisms into the human body resulting in health problems [1]. The number of bacterial infection cases is gradually increasing with the development of antimicrobial-resistant bacteria, including *Staphylococcus aureus*, *Streptococcus pneumonia*, *Acinetobacter baumannii*, *Escherichia coli*, *Klebsiella pneumoniae*, and *Pseudomonas aeruginosa* [2]. It has been estimated that 4.95 million people died from infections associated with bacterial resistance in 2019. Among those, 1.27 million deaths were predicted to directly result from antimicrobial resistance [3]. Different drug-resistant strains can develop through both natural and acquired resistance. The resistance mechanisms include (i) target modification, which leads to a decrease or loss of the affinity for the drug; (ii) inactivating enzyme that changes or destroys the drug; (iii) reduced permeability, which restricts the access of the drug into the cells; and (iv) efflux pump that expels the drug from the cell [4]. Several strategies have been established to address antimicrobial resistance (AMR), such as enhancing awareness of AMR, reducing infection incidence, and optimizing antimicrobial agent use [5]. *S. aureus* is a prominent human pathogen that causes mild to severe, life-threatening skin and soft tissue diseases, bacterial endocarditis, and infections associated with medical devices [6]. This microorganism is a harmful pathogen because of its great ability to become antibiotic resistant. The World Health Organization (WHO) has recently divided antibiotic-resistant bacteria into three categories (critical, high, and medium priority) according to the severity of the illness they cause, their resistance to many antibiotics, and the duration of treatment in the hospitals [7]. Methicillin-resistant *S. aureus* (MRSA) is listed as a highly prioritized pathogen, and the WHO encourages researchers to search for novel antibiotics that target this pathogen [8].

Antibiotics treat certain types of bacterial infections by either killing the bacteria that cause these infections or preventing their spread. Antibiotic drugs are natural secondary metabolites or chemically synthesized analogs produced by microorganisms. Most natural antibiotics are isolated from soil microorganisms that show phylogenetic diversity. The geographic region (soil depth and vegetation type) also affects the genetic potential for producing these metabolites [9]. Our previous study isolated several soil bacteria from the botanical and national parks in Thailand, and some of them showed potential antibacterial activity [10]. *Brevibacillus* sp. SPR20 (referred to as SPR20) exhibited the highest antibacterial activity at 24 h of incubation. This isolate produced effective antimicrobial peptides against the sensitive and resistant strains of *S. aureus*. Interestingly, these active compounds were stable in various conditions, such as temperature, pH, surfactants, and enzymes with a residual activity level of more than 90% [11].

Generally, the production of bioactive substances is greatly limited by biosynthetic gene clusters. Strain improvement by mutating the microbial genomes is important for biotechnology, food science, and biomanufacturing [12]. Physical and chemical mutagens are routinely used to alter nucleotides along the DNA strands. These techniques are particularly useful when the genetic information regarding the biosynthetic pathway is either limited or completely unknown. Genetic variation in the mutants results in a shift or bypass of the cellular regulatory controls, especially those in the key biosynthetic enzymes. This evolutionary process renders the mutated cells with desirable phenotypes, leading to the overproduction of desired bioactive substances [13]. Atmospheric and room temperature plasma (ARTP) is the latest physical approach to initiating random mutagenesis for microbial strain improvement [14]. ARTP is based on the ionization of working gas (argon or helium) by a radio-frequency electric field at atmospheric pressure. The generated plasma is blown through a nozzle onto a microbial sample. The generation of reactive chemical species (^•^OH, ^•^O_2_^−^, O_3_) is postulated to alter the microbial genome. The prokaryotic DNA damage is incompletely repaired, resulting in mutational accumulation. For example, *Bacillus subtilis* was exposed to helium plasma for 24 s, and a mutant was found to increase the yield of surfactin (a lipopeptide antibiotic) by 34.2% [15]. This mutated strain was genetically stable for ten generations and would be helpful in the industrial production of surfactin. Wang et al. (2010) used a helium plasma jet for 3 min to induce the mutations in *Streptomyces avermitilis* to increase the production of avermectins (antiparasitic agents) [16]. It caused a positive mutation rate of 21%, and the total avermectins and avermectin B1a were 18% and 43% higher than those in the wild-type strain, respectively. Therefore, ARTP is simpler and safer than other methods (UV radiation or nitrous acid). This technology generates a higher mutation rate and is widely used for microbial breeding [17]. 

This study aimed to improve the anti-MRSA activity of SPR20 using argon-based ARTP mutagenesis. The positive mutants were evaluated for their activities and genetic stabilities compared to the wild-type strain. Cell morphology, antibiotic susceptibility testing, and proteomic analysis were used to investigate the consequence of microbial mutagenesis. 

## 2. Results

### 2.1. ARTP Mutagenesis on SPR20

**Figure 1:**
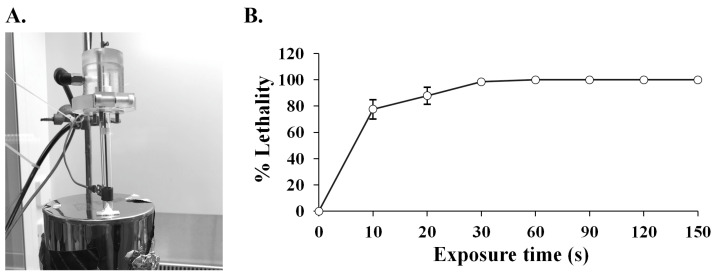
(**A**) Argon-based ARTP generator. It was constructed by plasma scientists at the Center of Excellence in Plasma Science and Electromagnetic Waves at Walailak University, Thailand. (**B**) Lethality of SPR20 after ARTP treatment. The percentage of lethality was determined at each point of different exposure times. Three replicates were performed for each experiment. The result is represented as mean ± SD (*n* = 3).

The argon-based plasma was applied to the cell suspension of SPR20 at different time intervals (Figure 1A). The result showed that a longer exposure time caused a higher cell lethality (Figure 1B). The lethality was substantially higher when the exposure time increased from 0 to 60 s: the percentage of lethality was 73.78 ± 7.35, 87.45 ± 6.46, 99.85 ± 0.33, and 99.99 ± 0.00 at 10, 20, 30, and 60 s, respectively. The repair mechanism in the bacterial cells was activated to compensate for DNA damage under this plasma radiation. The lethality was constant at 100.00 ± 0.00 percent when SPR20 encountered the ARTP jet in the period ranging from 90 to 150 s, indicating that the bacterial cells were completely killed (Figure 1B). This phenomenon may indicate that a large number of DNA lesions occurred and were beyond the DNA repair capacity, which resulted in cell death. Generally, the plasma radiation that caused a mortality rate of more than 90 percent was used for strain improvement because the higher positive mutations were found and exhibited the desired result [15,18]. This study selected 30 s as the treatment time because it was the shortest duration, corresponding to the optimal lethality.

### 2.2. Screening for the Positive Mutations

**Figure 2:**
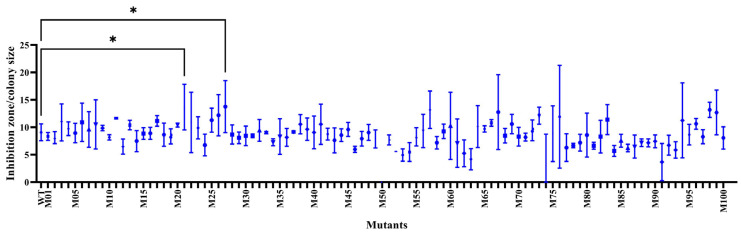
The diameter ratio between the inhibition zone and spot colony of the mutant and wild-type isolates against MRSA isolate 2468. * indicates a significant difference compared to the wild-type strain, as shown by the Student’s *t*-test at the *p*-value of less than 0.05.

A number of the survived SPR20 (100 isolates) were randomly evaluated for antibacterial activity against MRSA isolate 2468 using an agar overlay assay. The diameter ratio between the inhibition zone and spot colony of the mutants was compared to that of the wild-type strain, which was 9.08 ± 1.53. The result showed that there were 40 mutant isolates, giving a higher ratio from 9.16 ± 0.22 to 13.77 ± 4.74. It indicated that the ARTP procedure yielded a positive mutation rate of 40%, with M21 and M27 exhibiting a significantly higher ratio of 13.69 ± 4.15 and 13.77 ± 4.74, respectively (Figure 2 and Figure S1). 

### 2.3. Determination of the Antibacterial Activity of the Selected SPR20 Mutants

**Figure 3:**
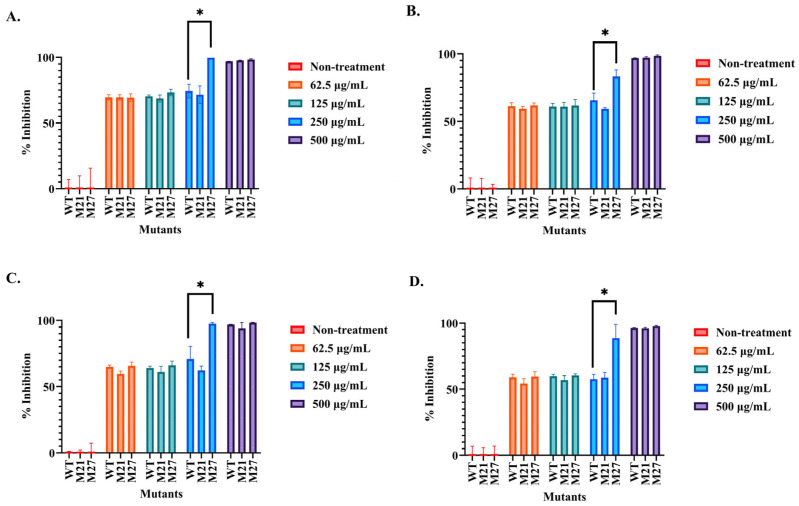
Microdilution assay of the mutants and the wild-type strain of SPR20. The samples from these strains were prepared in concentrations of 62.5, 125, 250, and 500 μg/mL. They were incubated with the culture of tested bacteria: (**A**) *S. aureus* TISTR 517 and (**B**–**D**) MRSA isolate 142, 1096, and 2468, respectively. The percentage of cell inhibition was measured based on the absorbance at 625 nm. * indicates a significant difference compared to the wild-type value, as shown by the Student’s *t*-test at the *p*-value of less than 0.05.

The mutant (M21 and M27) and wild-type SPR20 samples were prepared in the concentrations of 62.5, 125, 250, and 500 μg/mL and evaluated for antibacterial activity against tested bacteria by microdilution assay (Figure 3A–D). The result revealed that the samples (500 μg/mL) of M21, M27, and the wild-type strain could inhibit all tested bacteria by more than 94.01 ± 4.39% compared to the nontreatment condition. These samples in the 2-fold diluted concentrations between 62.5 and 250 μg/mL still inhibited the growth of all tested bacteria by more than 54.24 ± 3.78%. Interestingly, the sample of M27 at the concentration of 250 μg/mL exhibited significant inhibitory activity in the range from 83.41 ± 4.61% to 99.75 ± 0.00% when compared to those of the wild-type (57.66 ± 3.52% to 74.40 ± 5.18%) and M21 (58.84 ± 3.86% to 71.63 ± 6.69%) strains. The samples of M21 and M27 at the diluted concentrations (62.5 and 125 μg/mL) insignificantly inhibited the tested bacteria (54.24 ± 3.78% to 73.25 ± 2.35%) when compared to the wild-type strain at the corresponding concentration (59.08 ± 2.18% to 70.46 ± 0.98%). M27 showed the minimum inhibitory concentration (MIC) in the range of 250–500 μg/mL, whereas M21 and wild-type strains had an MIC value of 500 μg/mL. Therefore, M27 exhibited improved anti-MRSA activity after ARTP mutagenesis and was used in further investigations.

### 2.4. Comparison of Growth Curve and Antibacterial Activity

**Figure 4:**
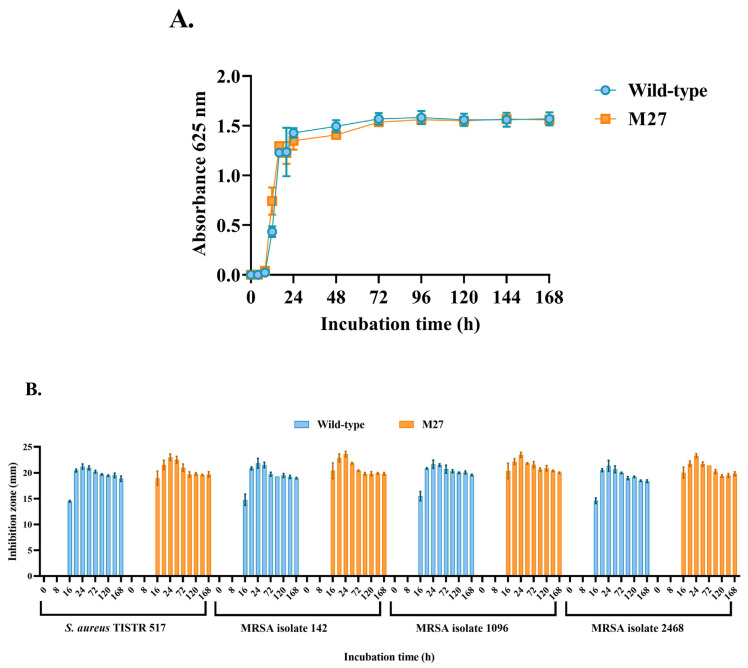
(**A**) Growth curve of the mutant (M27) and wild-type SPR20. (**B**) Inhibition zone of the CFS from M27 and wild-type strains.

The consequence of mutagenesis could enhance the bacterial growth rate and influence antibacterial activity [15,18]. The growth curves between the M27 and wild type were investigated. The result revealed that their growth curves were similar, and no significant difference in the growth occurred in these strains (Figure 4A). The lag phase of M27 and of the wild type was found within 12 h of incubation. These strains entered the exponential and stationary phases during 12–48 h and 48–168 h of incubation, respectively. The M27 and wild type produced the anti-MRSA substances in the cell-free supernatant (CFS), showing the highest activity against all tested pathogens at 24 h, which was associated with the exponential phase of the cells (Figure 4B). M27 gave a higher inhibition zone than the wild type in this period. The antibacterial activity of substances from both strains decreased after 48 h, corresponding to the stationary phase of the cell growth. Collectively, the growth rate of both strains was similar, and M27 had high anti-MRSA activity.

### 2.5. Strain Stability of the Selected Mutant

**Figure 5:**
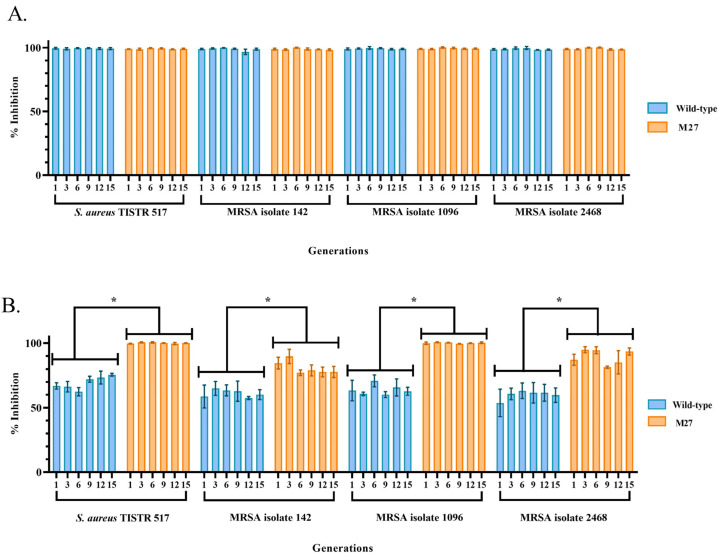

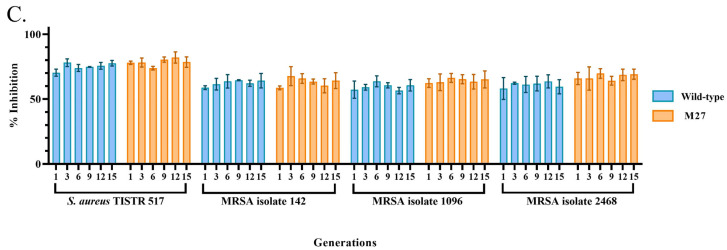
Determination of strain stability for fifteen generations. (**A**) The undiluted (500 μg/mL) and (**B**,**C**) diluted cell-free supernatant (250 and 125 μg/mL, respectively) of the M27 and wild type from different generations were used in the experiments. The percentage of cell inhibition was measured by a microdilution assay. * indicates a significant difference compared to the wild-type value, as shown by the Student’s *t*-test at the *p*-value of less than 0.05.

After sequentially subculturing M27 for fifteen generations (Generation 1, 3, 6, 9, 12, and 15), the inhibitory activities of the crude supernatant toward the tested bacteria were in the range from 97.43 ± 1.71% to 100.00 ± 0.26% at the initial concentration of 500 μg/mL (Figure 5A). A similar result of cell inhibition (96.66 ± 2.10% to 99.89 ± 0.15%) was found in the wild-type samples (500 μg/mL). These results showed no significant difference in the activity among generations. The activities of the diluted samples of the M27 and wild type in the concentrations from 125 to 250 μg/mL also had insignificant differences in their studied groups. However, the activity of the diluted samples was lower than that of the initial concentration (500 μg/mL) (Figure 5B,C). Interestingly, the inhibitory activity of M27 at 250 μg/mL (81.00 ± 5.60% to 100.14 ± 0.54%) was significantly higher than that of the wild type (60.11 ± 6.76% to 69.46 ± 5.45%), supporting the effect of mutagenesis on the increased activity of M27. Furthermore, all generations of M27 at 125 μg/mL had decreased anti-MRSA activities (63.11 ± 5.96% to 78.02 ± 4.59%), but they were not significantly different from those of the wild type (59.55 ± 4.22% to 75.09 ± 3.33%). It indicated that the M27 and wild type had MIC values of 250–500 and 500 μg/mL, respectively. These results supported the genetic stability of the M27 and wild type of SPR20 in the production of anti-MRSA substances.

### 2.6. Cell Morphology by Scanning Electron Microscopy (SEM) 

**Figure 6:**
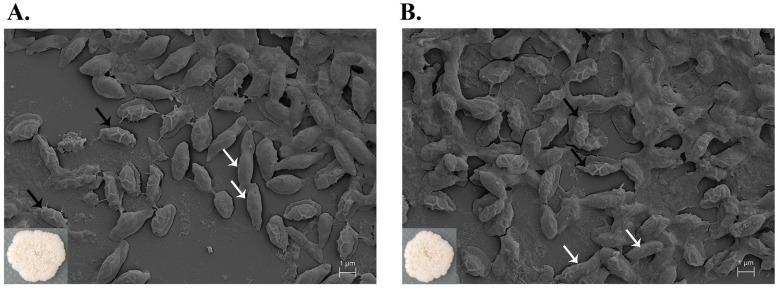
SEM micrograph of (**A**) M27 and (**B**) wild-type isolates of SPR20. The images were captured at the magnification of 50,000×. The white and black arrows presented the vegetative cells and their spores, respectively. The inset was the colony morphology on MH agar.

The M27 and wild-type strains displayed similar characteristics with circular and cream-colored colonies, showing a wrinkled surface and undulated margin. SEM micrographs showed that the vegetative cells of both strains had a similar rod shape with the dimensions of 3.26 ± 0.38 × 0.91 ± 0.03 µm and 2.92 ± 0.49 × 0.97 ± 0.13 µm for the M27 and wild type, respectively (Figure 6A,B). Their spores had rough and folded envelopes on the surface with the size of 2.24 ± 0.17 × 1.25 ± 0.16 µm and 2.40 ± 0.19 × 1.27 ± 0.17 µm. There was no significant difference in the cell dimension of the M27 and wild type, indicating that ARTP radiation did not affect the cell size.

### 2.7. Antibiotic Susceptibility Assay

Drug sensitivity of M27 and wild-type strains of SPR20 was compared by measuring the inhibition zone of antibiotic disks on the precultured MH agars. The result showed that M27 was susceptible to antibiotics that inhibited cell wall synthesis (cefoxitin, ceftriaxone, imipenem, piperacillin + tazobactam, and vancomycin) by causing the inhibition zone in the range from 21.08 ± 0.44 to 35.56 ± 0.25 mm (Table 1). The wild type was also sensitive to these drugs, causing a zone between 20.91 ± 0.96 and 36.41 ± 0.53 mm. The mutant and wild-type strains were also inhibited by the drugs that targeted protein synthesis (ciprofloxacin, doxycycline, erythromycin, and gentamicin) with zones of 26.66 ± 0.15 to 40.47 ± 0.29 and 26.49 ± 0.73 to 39.79 ± 0.78 mm, respectively. The sensitivity of M27 on these antibiotics was insignificantly different from the wild-type strain. 

### 2.8. Proteomic Variability by Principal Component Analysis

**Figure 7:**
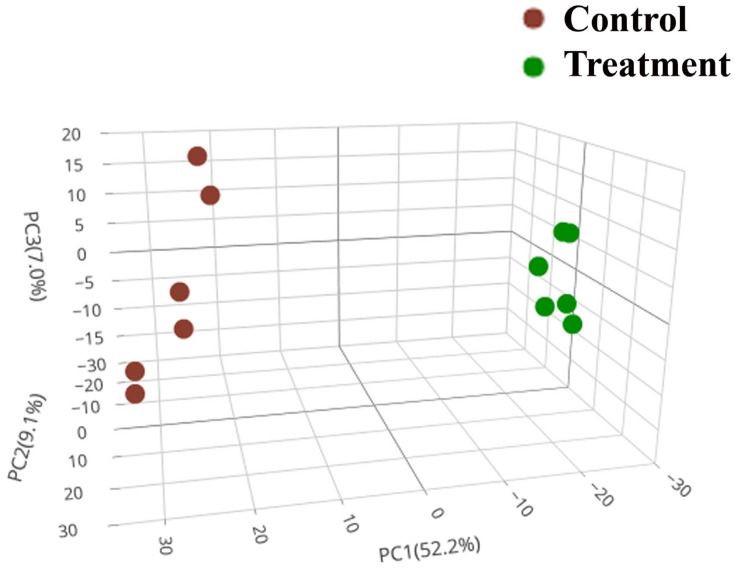
The 3D-PCA of the protein abundance of the treatment (M27) and control (wild-type) groups. The variances are presented in the brackets of the principal component axes. The different groups categorized by each component (PC1, PC2, and PC3) are displayed, in which the treatment and control groups are in green and red color, respectively.

Proteomic analysis was used to evaluate the extent of the changes in protein abundances and the level of variance in the proteome profiles. A principal component analysis (PCA) was performed to measure an overall proteomic variability between two conditions, enabling the effective differentiation of proteins obtained from the treatment (M27 mutant) and control (wild-type) groups. The result showed that the first principal component (PC1) displayed the most significant variance followed by the second and third principal components (PC2 and PC3), demonstrating the total variances of 52.2%, 9.1%, and 7.0%, respectively (Figure 7). The three-dimension PCA (3D-PCA) revealed the scattering distribution of dots, indicating substantial differences in the protein levels between the clusters of treatment and control groups.

### 2.9. Comparative Analysis of Proteomics

The complete proteome identification with the expression value was obtained by Orbitrap mass spectrometer. The proteomic data of 1645 proteins from 3 biological and 2 technical replicates (12 samples in total) are provided in Table S1. We performed the post-data processing to gain accurate protein lists for downstream analysis. The confidence level in the data was determined by important factors, such as mass deviation, false discovery rate (FDR), and digestion specificity. Maximum peptide mass variation was a key component for mass accuracy in the dataset [19]. The mass analysis showed that 89.27% of identified peptides had deviations of less than 10 ppm, implying that most peptides had excellent mass precision (Figure S2A). The small peptide mass deviation reduced the number of false-positive peptides. Most tryptic peptides (56.86%) contained 10–25 amino acids. Although miscleavage sites of tryptic peptides were occasionally observed in the proteomics analysis, the majority (81.74%) of all identified peptides exhibited no miscleavage sites, while 15.90% contained a single miscleavage site (Figure S2B). This demonstrates that our tryptic peptides exhibited a high specificity and similarity to the known attributes of the trypsin digestion method [20]. The assessment of protein identification quality in proteomics relies on the measurement of FDR. The target-decoy search is a standard method for FDR analysis, which gives a score to each peptide-spectrum match (PSM) based on the occurrence of false assignments [21]. In this analysis, we set an FDR threshold of less than 0.01 (1%) to ensure high confidence in the identified proteins. Subsequently, the data that met the qualification criteria were subjected to Venn diagram analysis and differential expression evaluation.

**Figure 8:**
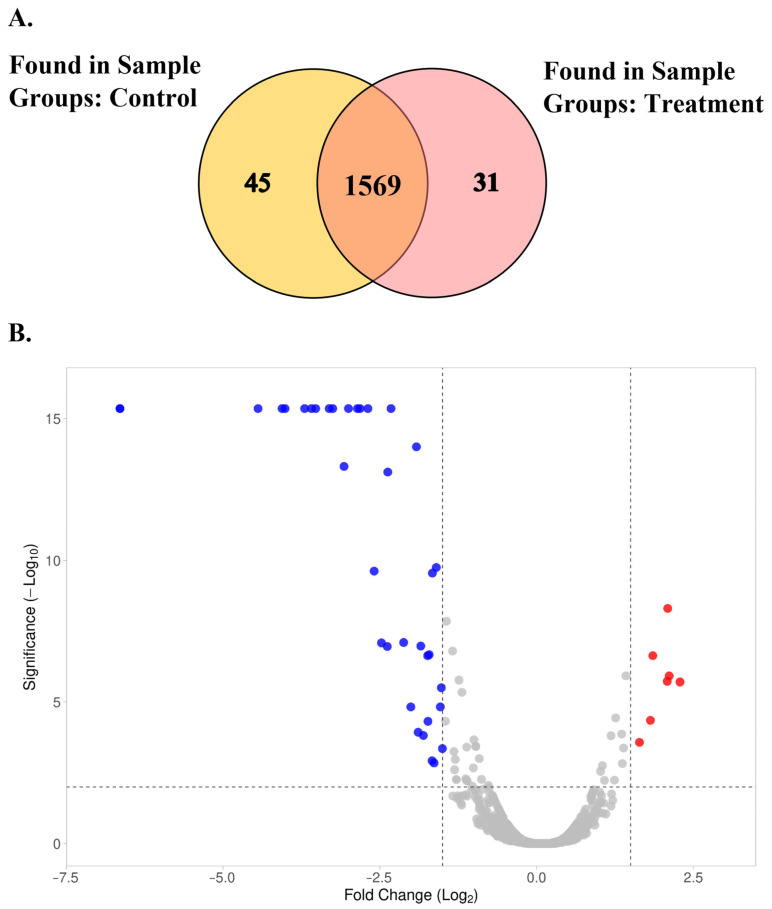
(**A**) The Venn diagram depicting the numbers of distinct and common proteins found in the treatment (M27 mutant) and control (wild-type) groups. The protein lists employed to construct this figure are provided in Table S1. (**B**) Quantitative proteomics analysis by volcano plot. The plot shows the negative log_10_ of the *p*-values plotted against the log_2_ of the fold change in each protein, comparing the treatment and control groups. Statistically significant proteins are plotted above the dash lines in red (*p* < 0.01) and blue (*p* < 0.01) dots for up- and downregulated expression upon plasma treatment.

Normalization of the label-free proteomics approach requires a dataset correction process. The profile spectra for different samples obtained from liquid-chromatography tandem mass spectrometry (LC-MS/MS) presented the intensity fluctuations of the peptide ions. To address this problem, protein normalization was performed to lower the variations associated with the non-biologics, making results reliable for downstream processing. The situation changed after normalization, and an interquartile range of total peptide abundances among all samples was approximately equivalent as shown in Figure S3. This normalization decreased the intragroup and intergroup variations measured as Log_10_ (protein abundance). The missing values in the raw data occur, particularly for components with relatively low abundance in the cellular system. It is a common phenomenon in LC-MS/MS due to the inherent randomness in sampling during the study [22]. These missing values can be addressed by either removing or including them, using a normalization algorithm and statistical analysis of all quantitated proteins. Based on the high-confidence protein data, a total of 1645 proteins were determined, exhibiting an estimated FDR close to 0 across all 12 LC runs. We identified the distinct and common proteins as illustrated in the Venn diagram. This diagram revealed 1569 proteins commonly found in both sample groups. There are 31 and 45 distinct proteins found in the mutant (treatment) and wild-type (control) strains of SPR20, respectively (Figure 8A). The unique proteins in the M27 mutant are summarized in Table 2. Based on the Venn diagram, the proteome profiling revealed differences in protein expressions between the M27 mutant and wild-type strains of SPR20 (Figure 8B). This volcano plot identifies the most significant changes in protein expression. Each spot represents the protein expression ratio between treatment and control groups according to their −log_10_ (*p*-value) and log_2_ (fold change). Notably, the volcano plot shows the significantly and differentially expressed proteins (*p*-value of less than 0.01 and −2 > log_2_ (fold change) > 2), and the comparative proteomics analysis shows that 13 and 36 proteins were upregulated (red dots) and downregulated (blue dots), respectively. The upregulated proteins in the M27 mutant were involved in metabolic processes (threonine synthase (*A0A385TD34*), ornithine aminotransferase (*rocD*), anthranilate synthase component II (*A0A0K9YU01*)), copper ion transport (*A0A075RAN9*), and components in the cell structures, such as flagellin (*A0A075R7M4*) and heptaprenylglyceryl phosphate synthase (*pcrB*). In contrast, the downregulated protein in M27 was implicated in metabolic processes (transposase (*V6MFL4*) and chitinase (*A0A177XMP1*)) and transporters of metal ion (*A0A075R420*) and oligopeptide (*M8DJR2*) (Table S1).

## 3. Discussion

Several studies have demonstrated the use of ARTP technology in improving microbial production of bioactive substances. The plasma produced by ionizing the working gas (argon and helium) under the electric field generates the reactive chemical species that cause DNA and protein damage. They postulated that plasma-induced cells activated repair enzymes to compensate for low or moderate DNA damage. However, the induction of the repair process was time-consuming, making it difficult to repair the severe DNA damage. This situation resulted in either an accumulation of mutations or cell death. Kurita et al. (2020) demonstrated that plasma radiation could generate reactive species and induce DNA modification (8-oxoguanine) and strand breaks in a cancer cell [23]. This cell also upregulated its corresponding repair enzyme, leading to a slight reduction in cell viability. The abundance of radical species primarily relies on the electric power and duration of treatment [24]. Due to the limitation of the high-voltage power supply, our study maintained a low input power (about 4 W), while varying the treatment time to promote optimal growth of the microbial strain without causing complete lethality. Our result was consistent with previous studies, in which the lethality was directly proportional to the treatment time closely related to the mutation rate. There was no cell survival after more prolonged exposure to plasma radiation at a specified time [25,26]. However, the effect of reactive species on bacterial DNA or proteins varied and rendered the different characteristics of mutants. Argon plasma could generate oxygen and nitrogen species, such as hydroxyl radicals (^•^OH), hydrogen peroxide (H_2_O_2_), or nitrite (NO^2−^), to damage the cells [27]. Various physical and chemical mutagens were used for microbial strain improvement. Huang et al. (2021) showed that ARTP produced greater DNA damage than UV radiation and 4-nitroquinoline-1-oxide or diethyl sulfate, respectively, in *Salmonella typhimurium* TA1535 [28]. The induction of β-galactosidase activity in this strain caused by ARTP was higher than that caused by UV, but its effect by chemical agents varied depending on the different treatment procedures (mutagenesis method, treatment duration, DNA repair ability) [28]. Our previous result revealed that the hydrogen peroxide yield increased according to the exposure time. The amount of nitrite decreased with treatment time, which might have resulted from nitrite conversion to nitrate [29]. Our study showed that a treatment time below 60 s could be employed for mutagenesis, but we opted for a shorter duration of 30 s because the lethality of more than 90% was a decisive factor in giving a highly positive mutation rate [15,18]. We found that the ARTP exposure time of 30 s yielded a positive mutation rate of 40%. Similarly, the treatment time of 3 min was selected in a study because it showed a desirable lethality rate of 98% on *Streptomyces avermitilis* with a positive mutation rate of 21% to produce avermectin [16]. Interestingly, iterative ARTP mutagenesis on *Streptomyces fradiae* improved the production of neomycin sulfate, in which the treatment time of 180 s was used for mutation induction as it caused the lethality of about 98%. The multiple rounds of mutagenesis gradually increased the potency of neomycin sulfate, in which the positive mutation was cumulative, with the rate of 5% and 60% after the first and sixth rounds of ARTP exposure [18]. Therefore, the plasma treatment offered numerous advantages, such as effective mutagenesis within a short period, operation in ambient air, and environmental friendliness [30]. The ARTP method had several limitations, including the need to screen a large number of surviving cells to identify enhanced antibacterial activity, the requirement to optimize the treatment time to determine the optimal condition, and the restriction of a small plasma volume, limiting its applicability to low sample volumes or surfaces.

Generally, plasma treatment mainly changes cell structures (pore formation and cell break) and cell growth. This structural change can be explained by the oxygen and nitrogen species induced by the plasma, promoting the oxidation of lipids and proteins of the cell membrane and causing various changes in cellular characteristics [31]. Interestingly, the SPR20 mutant had similar morphology and cell growth compared to its wild-type cells. This mutant presented excellent strain stability because of no significant difference in the antibacterial activity during the subsequent culture for fifteen generations. Likewise, ARTP mutagenesis on *Pseudomonas* sp. L01 generated a desired mutant with a similar growth rate and increased the yield rate of rhamnolipids (biosurfactants) after 36 h of incubation compared to the wild-type strain. The production of rhamnolipids was constant for five consecutive generations, indicating that the mutant was genetically stable. It suggested that plasma mutagenesis caused genetic mutations in the rhamnolipid biosynthesis but not in the cell growth and division [32]. In contrast, Gao et al. (2020) demonstrated that an ARTP-treated *Aspergillus oryzae* had a similar cell shape but exhibited a faster growth rate with more spores and mycelia when compared to its parental cell. The mutant increased the activity of salt-tolerant proteases and had excellent genetic stability after fifteen generations [33]. Additionally, a mutant of *Pseudomonas putida* KT2440 after ARTP treatment showed 5 times higher growth performance than the wild-type strain and enhanced the degradation capacity of 1,4-butanediol [34]. However, the different effects of plasma on bacterial cells might be due to the difference in external cell structure. Previous studies demonstrated that Gram-positive bacteria were more resistant to plasma than Gram-negative cells and showed no morphological changes after plasma treatment. Gram-positive species lacked an outer membrane but had a thicker murein layer, giving them higher strength and rigidity [31,35].

Mutation of bacterial DNA causes various genetic changes, making bacteria resistant to antibiotics. For example, the β-lactam antibiotics are potent inhibitors of transpeptidases or penicillin-binding proteins (PBPs) that catalyze the formation of peptidoglycan. These drugs inhibit the biosynthesis of bacterial cell walls, leading to bacterial lysis. Mutations of PBPs, such as Gln552Glu in *Streptococcus pneumoniae* PBP2x, reduce the affinity for the β-lactams and increase drug concentrations required for bacterial inhibition [36]. The substitution with the negative charge of glutamate is proposed to repel the negative charge of the β-lactam, decreasing the acylation efficiency for penicillin G and cefotaxime and, thereby, leading to drug resistance in this pathogen. Furthermore, DNA topoisomerase is important for bacterial DNA synthesis. Mutations in this gene usually cause amino acid substitutions, such as Ser83Leu and Asp87Asn mutations in *Escherichia coli* topoisomerase II, interrupting the water-metal ion bridge, thereby lowering the drug affinity of the enzyme-DNA complex. These mutations are associated with the increased MIC value, providing bacterial resistance to fluoroquinolones [37]. Random ARTP mutagenesis can promote resistance of the treated strains to antibiotics, and it is necessary to evaluate this further. The result from the antibiotic susceptibility assay revealed that the ARTP-treated SPR20 had no significant difference in drug sensitivity (β-lactams, fluoroquinolones, tetracyclines, macrolides, and aminoglycosides) compared to the wild-type species. The limitation of this study is the identification of precise mutations by genomic sequencing to understand the genetic mechanisms behind antibiotic susceptibility associated with target modification, enzymatic inactivation, and drug transport [38]. Our result might imply that the SPR20 mutant did not acquire a severe mutation accumulation to evolve drug resistance.

Recently, we identified five antimicrobial peptides from SPR20. These active peptides contained 14 or 15 amino acids and were cationic substances, resulting from the presence of lysine [11]. These valine-rich peptides contributed to the hydrophobicity of the molecules. BrSPR20-P1 (NH_2_-VVVNVLVKVLPPPVV-COOH) had an amphipathic structure and exhibited the highest anti-MRSA activity, promoting membrane permeation, pore formation, and cell lysis. The effect of ARTP mutagenesis on the enhanced antibacterial activity of SPR20 needs further investigation. Proteomic analysis showed that the plasma-treated cell (M27) produced some proteins, which were specifically found in the mutant and significantly upregulated the expression, such as threonine synthase, ornithine aminotransferase, and anthranilate synthase component II. The biological functions of these proteins were involved in the metabolic processes. Threonine synthase involves the elimination of phosphate from L-phosphohomoserine to produce L-threonine. Ornithine aminotransferase catalyzes the interconversion of ornithine to glutamate semialdehyde, affecting the L-glutamate and L-proline biosynthesis [39]. These proteins are catabolic enzymes that produce amino acids that are primary metabolites for cell survival, growth, and peptide or protein production [40]. Anthranilate synthase component II catalyzes chorismate to anthranilate, using glutamine as the source of the amino group and functions in glutamine metabolism [41]. Anthranilate is an important intermediate in the biosynthesis of indole, such as tryptophan. Anthranilate could inhibit biofilm formation in a broad range of bacteria (*S. aureus*, *B. subtilis*, and *P. aeruginosa*) [42]. Tryptophan-rich antimicrobial peptides were reported to kill bacteria by targeting cell membranes and intracellular molecules [43]. The upregulated expression of polyketide synthase was also found in the SPR20 strain after ARTP exposure. Polyketide synthases are the enzyme complexes that produce important secondary metabolites, such as tetracycline and macrolide antibiotics. Brevibacillin, an antimicrobial lipopeptide from *Brevibacillus laterosporus* OSY-I1, was produced from the activation of gene clusters, including polyketide synthases [44]. The compounds (auriporcine and basiliskamide) from *Brevibacillus latersporus* PE36 were active against MRSA and might be produced from polyketide synthase gene clusters [45]. Therefore, we postulated that the upregulated unique proteins in the mutant strain led to an increase in amino acids (proline and glutamate), and the catalytic enzymes simultaneously facilitated the synthesis of antimicrobial substances. BrSPR20-P1, which consisted of proline (20%), might potentially be one of the active substances in SPR20 [11].

ARTP mutagenesis is a powerful technique for generating SPR20 strains with desired random mutations that confer improved characteristics on antimicrobial activity. The consequence of the mutations might affect the key bacterial enzymes that promote the synthesis and secretion of the active substances. Activation of the silent or weakly expressed genes (cryptic biosynthetic gene cluster) might occur after mutagenesis, leading to the production of new antimicrobial agents [46]. Xu et al. (2021) investigated the whole genome sequencing of ARTP-treated *Bacillus subtilis* CICC 10721. It revealed that the mutations were found in the genes involved in the synthesis of amino acids and components of cell structures (cell membrane and movement) that resulted in the increased production and an accelerated efflux process of surfactin [15]. Furthermore, ARTP treatment on an inactive *Streptomyces* strain generated active mutants with antibacterial activity. Liu et al. (2021) performed genomic and metabolomic analyses to screen the active molecules. Aborycin was found and postulated to be a product of awakening the cryptic biosynthetic gene cluster induced by ARTP [47]. Our work obtained an SPR20 mutant with improved antibacterial activity. The comparative proteomic analysis showed that the mutant had high-level expressions of proteins implicated in amino acid metabolism, cell structures, and catalytic enzymes to produce active secondary metabolites. However, this study had a limitation on the assignment of the potential molecules, which might be known substances and novel cryptic antimicrobial agents. Whole genome sequencing is useful for the next study to define the exact gene alteration after ARTP exposure and confirm their correlations between potential key genes and proteomic profilings that support the enhanced antibacterial activity of plasma-treated mutants. 

## 4. Materials and Methods

### 4.1. Microorganisms and Culture Conditions

*Brevibacillus* sp. SPR20 (GenBank accession no. MN533919) was isolated from soil in a botanical park [10]. It was cultured on Mueller Hinton (MH) agar (Titan Biotech Ltd., Bhiwadi, India) and incubated at 30 °C for 24 h. *Staphylococcus aureus* TISTR 517 was purchased from the Thailand Institute of Scientific and Technological Research. Methicillin-resistant *S. aureus* (MRSA) clinical isolates 142, 1096, and 2468 were from a hospital in Nakhon Si Thammarat, Thailand. These tested bacteria were streaked on MH agar and incubated at 37 °C for 24 h. 

### 4.2. Atmospheric and Room Temperature Plasma (ARTP) Mutagenesis

Cell suspension of SPR20 was prepared by inoculating its single colony in 0.85% NaCl until the absorbance at 625 nm reached 0.60. An aliquot of cell suspension (10 μL) was transferred into a sterilized cap of polymerase chain reaction tube. The sample was subjected to a plasma nozzle by adjusting the distance between the sample and the nozzle to 2 mm. The input power amounted to approximately 4 W. Argon was employed as the working gas, flowing at a rate of 10 standard liters per minute (SLPM). The exposure time under the plasma jet was different (10, 20, 30, 60, 90, 120, and 150 s). The sample was subsequently diluted with 0.85% NaCl, and 100 μL of the treated sample was spread on MH agar to count the surviving cells. The experiments were performed in triplicate, and the untreated samples were used as the control groups. The lethality under different exposure times was obtained by

$$\mathrm{Lethality}\;(\%)=(\frac{U\;-T}{U})\times \;100$$

where T and U were total colony counts in the treated and untreated samples, respectively [18,29].

### 4.3. Mutant Screening by Agar Overlay Method

The single colonies of the SPR20 mutants after ARTP treatment were spotted on MH agar and incubated at 30 °C for 24 h. The cell suspension of MRSA isolate 2468 was prepared by inoculating the tested cells in 0.85% NaCl and adjusting the absorbance at 625 nm to 0.1. One mL of the cell suspension was added to 9 mL of melted soft MH agar (0.75% agar). This soft agar was poured over the surface of the SPR20-spotted plates and allowed to solidify for 1 h. The plates were incubated at 37 °C for 24 h. The vernier caliper was used to measure the inhibition zone observed around the mutant isolates. Subsequently, the diameter ratio between the inhibition zone and spot colony of SPR20 mutants was calculated. The wild-type strain was used as a control. The positive mutation rate was calculated by the following equation.

$$\mathrm{Positive}\;\mathrm{mutation}\;\mathrm{rate}\;(\%)=(\frac{A}{B})\;\times \;100$$

where A is the total colony count of mutants, which has a higher diameter ratio between the inhibition zone and spot colony than that of the wild-type, and B is the total colony count of all mutants [18]. 

### 4.4. Antibacterial Testing by Broth Microdilution Assay

The single colonies of the tested bacteria (*S. aureus* TISTR 517, MRSA isolate 142, 1096, and 2468) were cultured in MH agar at 37 °C for 16 h. The tested suspension of the bacteria was diluted by 0.85% NaCl until their absorbance was 0.10 at 625 nm. The suspension was subsequently diluted by cation-adjusted Mueller–Hinton broth (CAMHB) to obtain the cell concentration of 5 × 10^6^ CFU/mL. The SPR20 mutants with significantly higher activity than the wild type were inoculated in 0.85% NaCl, and their turbidities were adjusted until the absorbance of 0.10 at 625 nm was obtained. An amount of 1 mL of the diluted mutant cells was transferred to 49 mL of Luria Bertani (LB) broth, and the samples were incubated at 30 °C, 150 rpm for 24 h. The cell-free supernatant (CFS) was collected by centrifugation at 10,000× *g*, 4 °C for 15 min and then lyophilized by a freeze-drying apparatus (Christ, Martin Christ Gefriertrocknungsanlagen GmbH, Osterode am Harz, Germany). A total of 1 mg of the sample was reconstituted with CAMHB (1 mL), and the stock sample solution was prepared in a 2-fold serial dilution with CAMHB. Sample solutions in the amount of 100 μL (62.5, 125, 250, and 500 μg/mL) were transferred to the 96-well plates. In total, 10 μL of the tested cells was added to each well, and the plates were incubated at 37 °C for 24 h [48]. The sample of the wild-type strain and CAMHB without inoculum were used as the control and blank, respectively. The absorbance was measured by a microplate reader at 625 nm, and the percentage of cell inhibition was obtained by

$$\mathrm{Inhibition}\;(\%)\;=\;(1\;-\;\frac{A_{\mathrm{sample}}-A_{\mathrm{blank}}}{A_{\mathrm{control}}-A_{\mathrm{blank}}})\times \;100$$

where A_sample_ and A_control_ are the absorbances of samples in the wells of mutant and wild-type strains, respectively. A_blank_ is the absorbance of CAMHB alone.

### 4.5. Growth Curve and Agar Well Diffusion Assay

The single colonies of the mutant and wild-type strain of SPR20 were cultured in LB broth at 30 °C, 150 rpm for 24 h. The cell suspension was adjusted to reach an absorbance of 625 nm to 0.1 using 0.85% NaCl as a diluent. The cell preparation (1 mL) was added to 49 mL of LB broth and incubated at 30 °C, 150 rpm. The samples were collected at 4, 8, 12, 16, 20, 24, 48, 72, 96, 120, 144, and 168 h, and the absorbance was measured at 625 nm using fresh LB broth as a blank. The CFS was obtained by centrifugation at 10,000× *g*, 4 °C for 15 min before further assays by agar well diffusion method. Briefly, 100 μL of samples was transferred to each well of MH agar that was spread with the tested strains (*S. aureus* TISTR 517 and MRSA isolate 142, 1096, and 2468). Fresh LB broth was used as a blank control. The plates were incubated at 37 °C for 24 h, and the inhibition zone was measured [29].

### 4.6. Stability of the Mutant Strains

The genetic stability of SPR20 mutants was demonstrated by streaking the selected mutants on MH agar, and the plate was incubated at 30 °C for 24 h. A single colony was subsequently transferred onto the fresh MH agar before being incubated under the same conditions [49,50]. It was successively cultured for fifteen generations. The mutants in each generation were inoculated in LB broth. The cell suspensions were prepared by adjusting the turbidity equivalently to the absorbance of 0.10 at 625 nm using 0.85% NaCl as a diluent. An aliquot of the diluted samples (2%) was transferred to 49 mL of LB broth. The culture was incubated at 30 °C, 150 rpm for 24 h, and the supernatants were collected by centrifugation at 10,000× *g* at 4 °C for 15 min before lyophilization. The samples were reconstituted with CAMHB, and the broth microdilution assay was performed as described above. The strain stability of the mutants was evaluated by comparing the percentage of cell inhibition against *S*. *aureus* TISTR 517 and MRSA isolates 142, 1096, and 2468.

### 4.7. Cell Morphology by Scanning Electron Microscope (SEM)

The mutant and wild-type strains of SPR20 were cultured on MH agar at 30 °C for seven days. The cells were fixed on a glass slide, using 2.5% glutaraldehyde in 0.1 M phosphate buffer pH 7.2 for 48 h. The cell samples were washed twice with 0.1 M phosphate buffer pH 7.2. They were dehydrated by a stepwise incubation with ethanol (20%, 40%, 60%, 80%, and 100%), in which each step was performed at room temperature for 30 min. The samples were completely dried using a critical point drying technique (Quorum Technologies Ltd., Lewes, UK). They were coated with gold before the cell morphology was captured at a magnification of 50,000× by SEM (Carl Zeiss, Oberkochen, Germany) [10].

### 4.8. Antibiotic Susceptibility Test

The selected mutant and wild-type strains of SPR20 were cultured in LB broth at 30 °C, 150 rpm for 24 h, and were diluted with 0.85% NaCl until the absorbance at 625 nm was equal to 0.10. A total of 100 μL of the diluted culture was spread on MH agar. The antibiotic disks (Oxoid Ltd., Basingstoke, UK) were placed on the surface of a pre-inoculated agar. The plates were then incubated at 30 °C for 24 h. The diameter of the inhibition zone was measured using a Vernier caliper [51].

### 4.9. Label-Free Quantitated Proteomics Analysis

To investigate the protein expression profiles of the mutant and wild-type strains of SPR20, the protein extraction was followed by a previous study [52]. Briefly, the cell pellets were resuspended in a lysis buffer (0.1x PBS buffer pH 7.4, 1% SDS, 5 mM DTT, and 1 mM PMSF) and lysed by an ultrasonic probe (Sonics VCX-500 processor, Sonics & Materials. Inc., Newtown, CT, USA) at 60% amplitude for 30 s. The lysed cells were centrifuged at 10,000× *g*, 4 °C for 20 min, and the proteins in the supernatant were precipitated using ice-cold acetone and stored at −20 °C for 16 h. The protein pellets were reconstituted in 0.25% RapiGest SF surfactant (Waters Corporation, Manchester, UK) in 10 mM ammonium bicarbonate. The protein concentration was measured by the Bradford test using bovine serum albumin as a standard. The total protein (25 µg) was subjected to trypsin digestion, in which the protein samples were reduced by utilizing 5 mM DTT in 10 mM ammonium bicarbonate at 90 °C for 15 min, and then the alkylation of sulfhydryl was performed at room temperature for 25 min in the dark. The solution was cleaned up by a Zeba-Spin desalting column (Thermo Fisher Scientific, Rockford, IL, USA). The flow-through solution was enzymatically digested by trypsin (Promega GmbH, Walldorf, Germany) at a ratio of 1:50 (enzyme: protein) and incubated at 37 °C for 6 h. The digested peptides were reconstituted in 0.1% formic acid and transferred to a TruView LCMS vial (Waters Corporation, Manchester, UK). A total amount of peptides (1.25 μg) was subjected to liquid-chromatography tandem mass spectrometry (LC-MS/MS). The spectrum data was collected in a positive mode on an Orbitrap HF hybrid mass spectrometer combined with an EASY-nLC1000 nano-LC system (Thermo Fisher Scientific, San Jose, CA, USA) with a nano C18 column. Mobile phase A (0.1% formic acid in water) and B (0.1% formic acid in 95% acetonitrile) were utilized in the LC condition as the following: the linear gradient from 2–45% B for 105 min, the regeneration at 90% B for 10 min, and the re-equilibration at 5% B for 35 min. The peptides were analyzed using a data-dependent acquisition method, followed by a higher-energy collisional dissociation at a collision energy of 28. A full-scan mass spectra (MS) were acquired from the mass-to-charge ratio (*m*/*z*) between 400 and 1600 with an automatic gain control (AGC) target at 3 × 10^6^ ions and a resolution of 120,000. MS/MS scan was initiated when the ACG target reached 10^5^ ions and a resolution of 15,000. The raw mass spectra were processed by Proteome Discoverer 2.4 and identified against the UniProt protein database (Organism: *brevibacillus* database; 195,729 sequences retrieved on 22 October 2021). To identify and quantify proteins, the following parameters were used: peptide tolerance (20 ppm), fragment tolerance (0.05 Da), minimum fragment ion matches per peptide (2), digest enzyme (trypsin), fixed modification of cysteine carbamidomethylation, and variable modifications of methionine oxidation. The false discovery rate (FDR) for both peptide and protein identification was set to 1%. The relative protein abundance ratio was normalized using the software’s normalization algorithm (total intensity count) by considering the total peptide amount across all LC runs (n = 12). The mass spectrometry proteomics data have been deposited to the ProteomeXchange Consortium via the PRIDE partner repository [53] with the dataset identifier PXD043954. 

### 4.10. Proteomics Data Analysis and Bioinformatics Analysis

The Proteome Discoverer software (version 2.4) utilized multiple consensus workflows to compile peptide-spectrum matches (PSMs) into peptide groups, protein database matches, and nonredundant protein groups to identify the differentially expressed protein list. This compilation was based on the principle of strict parsimony, as defined by the software’s default settings. The heterogeneity of variation in two experimental conditions was investigated using PCA to visualize differences among intra- and intergroup replicates. Microsoft Word 365 was used to create Venn diagrams that grouped the identified proteins from different species, highlighting both unique and common proteins. To ensure error-free log transformation for pathway over-representation analysis, all protein expression values were transformed into relative expression data by adding “1” to each expression value. A gene ontology enrichment analysis was manually performed using the web-based software tools, a Uniprot database (http://www.uniprot.org/uniprot) (accessed on 22 October 2021) [54].

### 4.11. Statistical Analysis

Three replicates were investigated in each experiment, and the result was shown as mean ± standard deviations (SD). The Student’s *t*-test or one-way analysis of variance (one-way ANOVA) was used to determine the significant difference at the *p*-value of less than 0.05. For proteomics analysis, one-way ANOVA was performed by Proteome Discoverer using a *p*-value of less than 0.05. Duncan’s multiple range test measured significant differences between pairs of means.

## 5. Conclusions

SPR20 was mutagenized by ARTP, and a high positive mutation rate (40%) with an enhanced anti-MRSA activity was obtained. The M27 mutant exhibited the most increased activity and excellent genetic stability across fifteen generations. This mutant showed insignificant changes in cell morphology, growth rate, and antibiotic susceptibility compared to the wild-type strain. Comparative proteomic analysis revealed that the plasma-exposed mutant had a higher expression of proteins associated with amino acid synthesis (threonine synthase and ornithine aminotransferase), cell structure (flagellin), and catalytic enzymes (polyketide synthase and anthranilate synthase component II). These unique proteins might support the increased antibacterial activity of mutants after ARTP treatment. ARTP leads to random mutagenesis throughout bacterial DNA, and the extent of DNA damage depends on the treatment duration. Further studies involving whole genome sequencing and identification of the active substances are necessary to elucidate the consequences of plasma. However, the mutated SPR20 will be a valuable resource for developing antibacterial agents, especially those for antibiotic-resistant infections. Additionally, this research will emphasize the effective ARTP method as a reference technique for microbial breeding. 

## Figures and Tables

**Table 1:** Susceptibility testing of M27 and wild-type strains of SPR20. Standard antibiotic disks were placed on the surface of SPR20-cultured MH agar. The diameters of the inhibition zone were determined and reported as mean ± SD (*n* = 3).

Antibiotics	Inhibition Zone (mm ± SD)	
	M27	Wild-Type
Cefoxitin (FOX; 30 µg)	33.02 ± 0.51	34.04 ± 0.00
Ceftriaxone (CRO; 30 µg)	32.07 ± 1.34	32.91 ± 0.78
Ciprofloxacin (CIP; 5 µg)	35.56 ± 1.02	35.56 ± 0.51
Doxycycline (DO; 30 µg)	40.47 ± 0.29	39.79 ± 0.78
Erythromycin (E; 15 µg)	36.91 ± 0.64	37.76 ± 0.39
Gentamicin (CN; 10 µg)	26.66 ± 0.15	26.49 ± 0.73
Imipenem (IPM; 10 µg)	35.05 ± 1.02	34.71 ± 0.29
Vancomycin (VA; 30 µg)	21.08 ± 0.44	20.91 ± 0.96
Piperacillin + Tazobactam (TZP; 110 µg)	35.56 ± 0.25	36.41 ± 0.53

**Table 2:** List of the unique proteins identified in M27 (treatment group). The information on protein descriptions and functions was obtained from https://www.uniprot.org accessed on 22 October 2021.

No.	Gene Name	Protein Name	Biological Process	Molecular Function
1	*pcrB*	Heptaprenylglyceryl phosphate synthase		
2	*A0A075RAN9*	Putative copper-importing P-type ATPase A	transport	catalytic activity; metal ion binding; nucleotide binding; transporter activity
3	*A0A2S5H3D0*	Multifunctional 2′,3′-cyclic-nucleotide 2′-phosphodiesterase/5′-nucleotidase/3′-nucleotidase		
4	*A0A385TD34*	Threonine synthase		
5	*A0A177XSD9*	Urocanate reductase	metabolic process	catalytic activity; nucleotide binding
6	*A0A075R7M4*	Flagellin	cellular component movement	structural molecule activity
7	*A0A0K9YU01*	Anthranilate synthase component II	metabolic process	catalytic activity
8	*A0A3M8C1C7*	Lipoprotein		
9	*glpX*	Fructose-1,6-bisphosphatase		
10	*J3AIB6*	Uncharacterized protein		
11	*rocD*	Ornithine aminotransferase		
12	*A0A4Y3PDX0*	Probable glycine dehydrogenase (decarboxylating) subunit 1		
13	*dut*	Deoxyuridine 5′-triphosphate nucleotidohydrolase		
14	*A0A177XIL4*	Polyketide synthase	metabolic process	catalytic activity
15	*ribH*	6,7-dimethyl-8-ribityllumazine synthase		
16	*A0A502H7N5*	3-ketoacyl-ACP reductase		
17	*A0A177XS18*	Carbonic anhydrase		catalytic activity; metal ion binding
18	*gatB*	Aspartyl/glutamyl-tRNA(Asn/Gln)-amidotransferase subunit B		
19	*deoD*	Purine nucleoside phosphorylase DeoD-type		
20	*A0A2S5HRX3*	Nickel ABC transporter substrate-binding protein		
21	*alaC*	Aminotransferase		
22	*A0A3M8D599*	Chromosome segregation protein SMC		
23	*pgk*	Phosphoglycerate kinase		
24	*accC*	Biotin carboxylase		
25	*yvdD*	Cytokinin riboside 5′-monophosphate phosphoribohydrolase		
26	*fusA*	Elongation factor G		
27	*rnr*	Ribonuclease R		
28	*A0A220MEP0*	Bacillithiol biosynthesis deacetylase BshB1	metabolic process	catalytic activity
29	*A0A075R422*	Dihydrolipoamide acetyltransferase component of pyruvate dehydrogenase complex	metabolic process	catalytic activity
30	*A0A075RA12*	General stress protein 14	metabolic process	catalytic activity
31	*A0A177XNX2*	ABC transporter ATP-binding protein		catalytic activity; nucleotide binding

## Data Availability

Not applicable.

## Supplementary Materials

The supporting information can be downloaded at: https://www.mdpi.com/article/10.3390/ijms241512016/s1.

## References

[1] Bloom D.E., Cadarette D. (2019). Infectious disease threats in the twenty-first century: Strengthening the global response. Front. Immunol..

[2] Zhu Y., Huang W.E., Yang Q. (2022). Clinical perspective of antimicrobial resistance in bacteria. Infect. Drug Resist..

[3] Antimicrobial Resistance Collaborators (2022). Global burden of bacterial antimicrobial resistance in 2019: A systematic analysis. Lancet.

[4] Munita J.M., Arias C.A. (2016). Mechanisms of antibiotic resistance. Microbiol. Spectr..

[5] Patel J., Harant A., Fernandes G., Mwamelo A.J., Hein W., Dekker D., Sridhar D. (2023). Measuring the global response to antimicrobial resistance, 2020–2021: A systematic governance analysis of 114 countries. Lancet Infect. Dis..

[6] Mancuso G., Midiri A., Gerace E., Biondo C. (2021). Bacterial antibiotic resistance: The most critical pathogens. Pathogens.

[7] Antimicrobial Resistance Division (2019). 2019 Antibacterial Agents in Clinical Development: An Analysis of the Antibacterial Clinical Development Pipeline.

[8] Terreni M., Taccani M., Pregnolato M. (2021). New antibiotics for multidrug-resistant bacterial strains: Latest research developments and future perspectives. Molecules.

[9] Sharrar A.M., Crits-Christoph A., Méheust R., Diamond S., Starr E.P., Banfield J.F. (2020). Bacterial secondary metabolite biosynthetic potential in soil varies with phylum, depth, and vegetation type. mBio.

[10] Songnaka N., Lertcanawanichakul M., Atipairin A. (2021). Promising anti-MRSA activity of *Brevibacillus* sp. isolated from soil and strain improvement by UV mutagenesis. Sci. Pharm..

[11] Songnaka N., Lertcanawanichakul M., Hutapea A.M., Krobthong S., Yingchutrakul Y., Atipairin A. (2022). Purification and characterization of novel anti-MRSA peptides produced by *Brevibacillus* sp. SPR-20. Molecules.

[12] Zhang Y.P., Sun J., Ma Y. (2017). Biomanufacturing: History and perspective. J. Ind. Microbiol. Biotechnol..

[13] Parekh S., Vinci V.A., Strobel R.J. (2000). Improvement of microbial strains and fermentation processes. Appl. Microbiol. Biotechnol..

[14] Wei X., Wang Y., Liu X., Hu Z., Qian J., Shi T., Wang Y., Ye C. (2023). Metabolic analysis of *Schizochytrium* sp. mutants with high EPA content achieved with ARTP mutagenesis screening. Bioprocess Biosyst. Eng..

[15] Xu H., Dai C., Tang Y., Xu X., Umego E.C., He R., Ma H. (2021). The selective breeding and mutagenesis mechanism of high-yielding surfactin *Bacillus subtilis* strains with atmospheric and room temperature plasma. J. Sci. Food Agric..

[16] Wang L.Y., Huang Z.L., Li G., Zhao H.X., Xing X.H., Sun W.T., Li H.P., Gou Z.X., Bao C.Y. (2010). Novel mutation breeding method for *Streptomyces avermitilis* using an atmospheric pressure glow discharge plasma. J. Appl. Microbiol..

[17] Ottenheim C., Nawrath M., Wu J.C. (2018). Microbial mutagenesis by atmospheric and room-temperature plasma (ARTP): The latest development. Bioresour. Bioprocess..

[18] Yu F., Zhang M., Sun J., Wang F., Li X., Liu Y., Wang Z., Zhao X., Li J., Chen J. (2022). Improved neomycin sulfate potency in *Streptomyces fradiae* using atmospheric and room temperature plasma (ARTP) mutagenesis and fermentation medium optimization. Microorganisms.

[19] Schulze W.X., Mann M. (2004). A novel proteomic screen for peptide-protein interactions. J. Biol. Chem..

[20] Lasch P., Schneider A., Blumenscheit C., Doellinger J. (2020). Identification of microorganisms by liquid chromatography-mass spectrometry (LC-MS) and in silico peptide mass libraries. Mol. Cell. Proteom..

[21] Gorshkov V., Hotta S.Y., Verano-Braga T., Kjeldsen F. (2016). Peptide *de novo* sequencing of mixture tandem mass spectra. Proteomics.

[22] Wei R., Wang J., Su M., Jia E., Chen S., Chen T., Ni Y. (2018). Missing value imputation approach for mass spectrometry-based metabolomics data. Sci. Rep..

[23] Kurita H., Haruta N., Uchihashi Y., Seto T., Takashima K. (2020). Strand breaks and chemical modification of intracellular DNA induced by cold atmospheric pressure plasma irradiation. PLoS ONE.

[24] Liu K., Xia H., Yang M., Geng W., Zuo J., Ostrikov K. (2022). Insights into generation of OH radicals in plasma jets with constant power: The effects of driving voltage and frequency. Vacuum.

[25] Zhang C., Qin J., Dai Y., Mu W., Zhang T. (2019). Atmospheric and room temperature plasma (ARTP) mutagenesis enables xylitol over-production with yeast *Candida tropicalis*. J. Biotechnol..

[26] Peng Q., Xiao Y., Zhang S., Zhou C., Xie A., Li Z., Tan A., Zhou L., Xie Y., Zhao J. (2022). Mutation breeding of *Aspergillus niger* by atmospheric room temperature plasma to enhance phosphorus solubilization ability. PeerJ.

[27] Wende K., Bekeschus S., Schmidt A., Jatsch L., Hasse S., Weltmann K.D., Masur K., von Woedtke T. (2016). Risk assessment of a cold argon plasma jet in respect to its mutagenicity. Mutat. Res. Genet. Toxicol. Environ. Mutagen..

[28] Huang Y., Wang L., Zhang X., Su N., Li H., Oda Y., Xing X. (2021). Quantitative evaluation of DNA damage caused by atmospheric and room-temperature plasma (ARTP) and other mutagenesis methods using a rapid *umu*-microplate test protocol for microbial mutation breeding. Chin. J. Chem. Eng..

[29] Songnaka N., Nisoa M., Atipairin A., Wanganuttara T., Chinnawong T. (2022). Enhanced antibacterial activity of *Brevibacillus* sp. SPR19 by atmospheric and room temperature plasma mutagenesis (ARTP). Sci. Pharm..

[30] Šimončicová J., Kryštofová S., Medvecká V., Ďurišová K., Kaliňáková B. (2019). Technical applications of plasma treatments: Current state and perspectives. Appl. Microbiol. Biotechnol..

[31] Niedźwiedź I., Juzwa W., Skrzypiec K., Skrzypek T., Waśko A., Kwiatkowski M., Pawłat J., Polak-Berecka M. (2020). Morphological and physiological changes in *Lentilactobacillus hilgardii* cells after cold plasma treatment. Sci. Rep..

[32] Xu Y., Jing Y., Zhang Q., Xiu J., Tian M., Cui Q., Ma Y., Yi L., Han L., Qian Y. (2023). Improving rhamnolipids biosynthesis in *Pseudomonas* sp. L01 through atmospheric and room-temperature plasma (ARTP) mutagenesis. Microorganisms.

[33] Gao X., Liu E., Yin Y., Yang L., Huang Q., Chen S., Ho C.T. (2020). Enhancing activities of salt-tolerant proteases secreted by *Aspergillus oryzae* Using atmospheric and room-temperature plasma mutagenesis. J. Agric. Food. Chem..

[34] Qian X., Xin K., Zhang L., Zhou J., Xu A., Dong W., Jiang M. (2023). Integration of ARTP mutation and adaptive laboratory evolution to reveal 1,4-butanediol degradation in *Pseudomonas putida* KT2440. Microbiol. Spectr..

[35] Laroussi M., Mendis D.A., Rosenberg M. (2003). Plasma interaction with microbes. New J. Phys..

[36] Mouz N., Di Guilmi A.M., Gordon E., Hakenbeck R., Dideberg O., Vernet T. (1999). Mutations in the active site of penicillin-binding protein PBP2x from *Streptococcus pneumonia*: Role in the specificity for beta-lactam antibiotics. J. Biol. Chem..

[37] Tang K., Zhao H. (2023). Quinolone antibiotics: Resistance and therapy. Infect. Drug Resist..

[38] Lopatkin A.J., Bening S.C., Manson A.L., Stokes J.M., Kohanski M.A., Badran A.H., Earl A.M., Cheney N.J., Yang J.H., Collins J.J. (2021). Clinically relevant mutations in core metabolic genes confer antibiotic resistance. Science.

[39] Richts B., Rosenberg J., Commichau F.M. (2019). A Survey of pyridoxal 5’-phosphate-dependent proteins in the Gram-positive model bacterium *Bacillus subtilis*. Front. Mol. Biosci..

[40] Liu Y.K., Kuo H.C., Lai C.H., Chou C.C. (2020). Single amino acid utilization for bacterial categorization. Sci. Rep..

[41] Romero R.M., Roberts M.F., Phillipson J.D. (1995). Anthranilate synthase in microorganisms and plants. Phytochemistry.

[42] Li X.H., Kim S.K., Lee J.H. (2017). Anti-biofilm effects of anthranilate on a broad range of bacteria. Sci. Rep..

[43] Mishra A.K., Choi J., Moon E., Baek K.H. (2018). Tryptophan-rich and proline-rich antimicrobial peptides. Molecules.

[44] Miljkovic M., Jovanovic S., O’Connor P.M., Mirkovic N., Jovcic B., Filipic B., Dinic M., Studholme D.J., Fira D., Cotter P.D. (2019). *Brevibacillus laterosporus* strains BGSP7, BGSP9 and BGSP11 isolated from silage produce broad spectrum multi-antimicrobials. PLoS ONE.

[45] Theodore C.M., Stamps B.W., King J.B., Price L.S., Powell D.R., Stevenson B.S., Cichewicz R.H. (2014). Genomic and metabolomic insights into the natural product biosynthetic diversity of a feral-hog-associated *Brevibacillus laterosporus* strain. PLoS ONE.

[46] Hoskisson P.A., Seipke R.F. (2022). Cryptic or silent? The known unknowns, unknown knowns, and unknown unknowns of secondary metabolism. mBio.

[47] Liu T., Huang Z., Gui X., Xiang W., Jin Y., Chen J., Zhao J. (2021). Multi-omics comparative analysis of *streptomyces* mutants obtained by iterative atmosphere and room-temperature plasma mutagenesis. Front. Microbiol..

[48] Clinical and Laboratory Standards Institute (2018). Methods for Dilution Antimicrobial Susceptibility Tests for Bacteria That Grow Aerobically.

[49] Zhang B., Zhang H.D., Zhou Y.T., Huang K., Liu Z.Q., Zheng Y.G. (2018). Improvement of amphotericin B production by a newly isolated *Streptomyces nodosus* mutant. Biotechnol. Appl. Biochem..

[50] Benkova M., Soukup O., Marek J. (2020). Antimicrobial susceptibility testing: Currently used methods and devices and the near future in clinical practice. J. Appl. Microbiol..

[51] Jorgensen J.H., Ferraro M.J. (2009). Antimicrobial susceptibility testing: A review of general principles and contemporary practices. Clin. Infect. Dis..

[52] Krobthong S., Choowongkomon K., Suphakun P., Kuaprasert B., Samutrtai P., Yingchutrakul Y. (2021). The anti-oxidative effect of Lingzhi protein hydrolysates on lipopolysaccharide-stimulated A549 cells. Food Biosci..

[53] Perez-Riverol Y., Bai J., Bandla C., García-Seisdedos D., Hewapathirana S., Kamatchinathan S., Kundu D.J., Prakash A., Frericks-Zipper A., Eisenacher M. (2022). The PRIDE database resources in 2022: A hub for mass spectrometry-based proteomics evidences. Nucleic Acids Res..

[54] UniProt Consortium (2021). UniProt: The universal protein knowledgebase in 2021. Nucleic Acids Res..

