# Supplementary material for: Atmospheric and Room Temperature Plasma (ARTP) Mutagenesis Improved the Anti-MRSA Activity of Brevibacillus sp. SPR20
Source: Int J Mol Sci. 2023 Jul 27;24(15):12016. doi: 10.3390/ijms241512016 (PMC10419081; doi:10.3390/ijms241512016)

Supplementary Materials: Atmospheric and Room Temperature Plasma (ARTP) Mutagenesis Improved the Anti-MRSA Activity of *Brevibacillus* sp. SPR20

Nuttapon Songnaka, Monthon Lertcanawanichakul, Albert Manggading Hutapea, Mudtorlep Nisoa, Sucheewin Krobthong, Yodying Yingchutrakul and Apichart Atipairin

Figure S1. Agar overlay assay of SPR20 mutants and wild-type. The colonies of (A) M21–M25 and (B) M26–30 were spotted before testing against MRSA isolate 2468. The wild-type colony was also cultured as a control.

A. B.

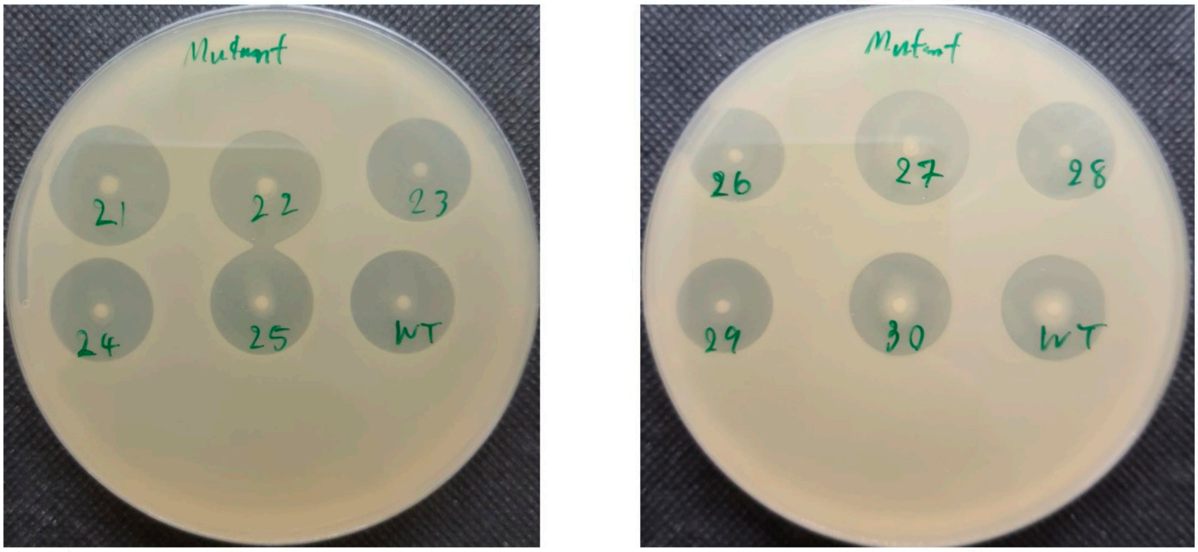

Figure S2. Post-data processing of LC-MS/MS dataset. (A) Distribution of delta mass of the peptides. Delta mass was the deviation of the measured mass from the theoretical mass of the peptide shown in the ppm unit. (B) Percentage of identified peptides with the missed tryptic cleavage sites.

A.

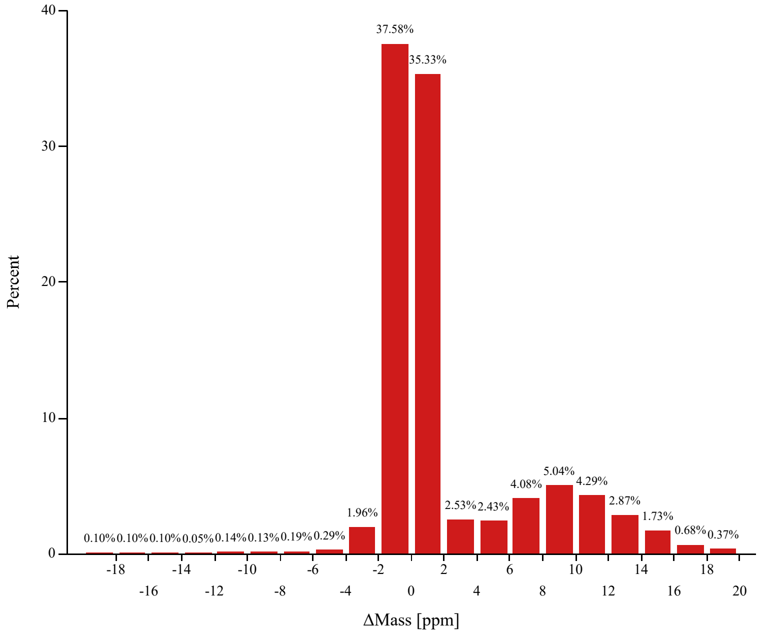

B.

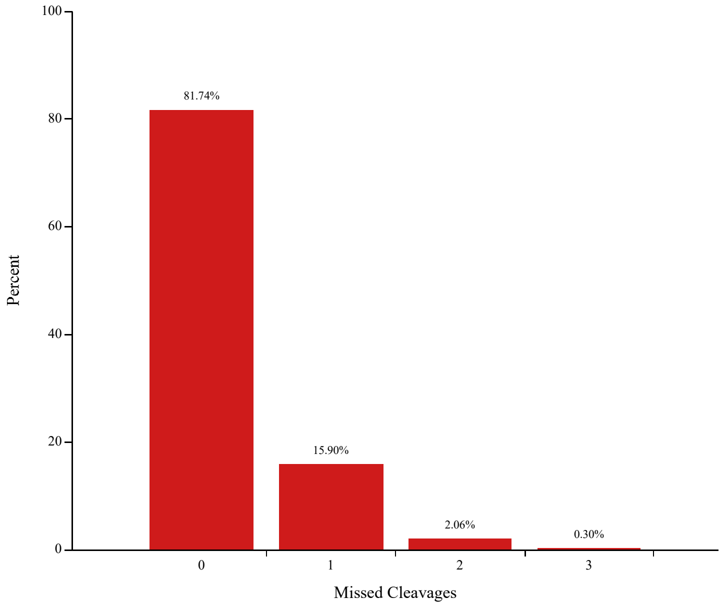

Figure S3. Protein level normalization. Y-axis was the Log_{10} scale of protein abundance, and X-axis was the samples of treatment and control groups.

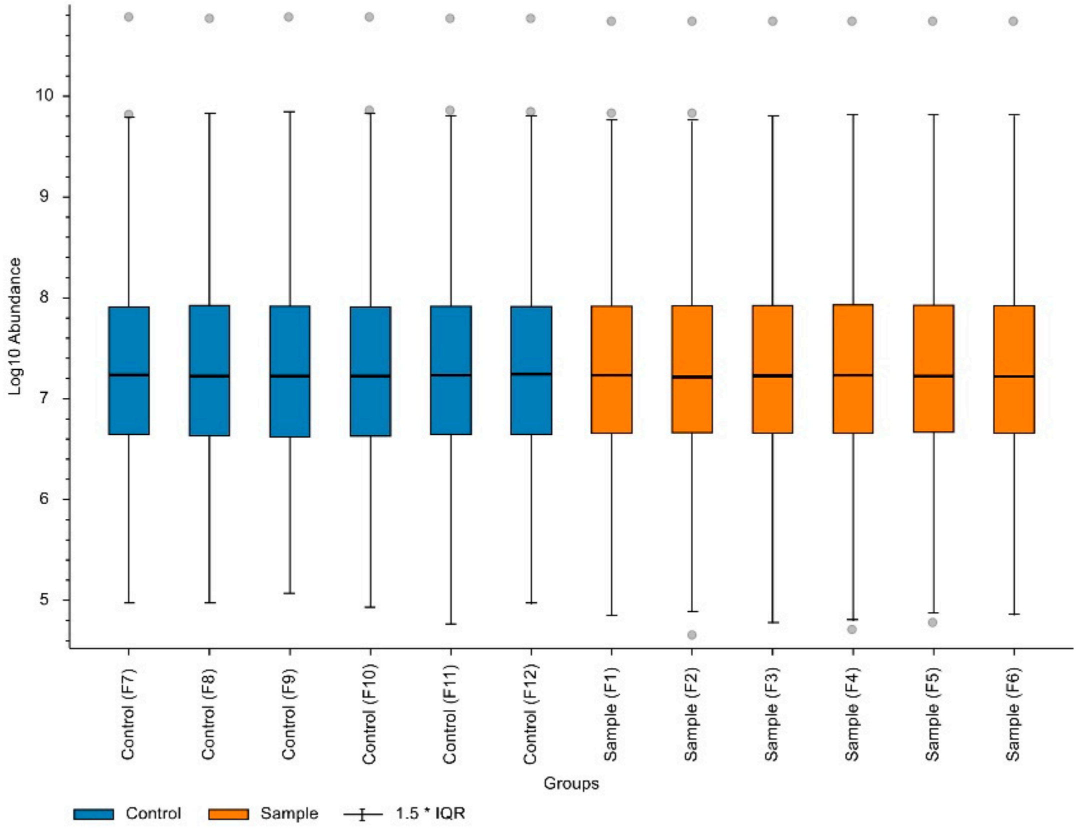

Supplement: Supplementary file 1 [file ijms-24-12016-s001.zip › 2 - Supplement Material Figure.pdf]
